# Lessons from surgery after neoadjuvant chemoimmunotherapy for bulky, invasive N2 lung cancer: a case report

**DOI:** 10.1186/s44215-026-00271-4

**Published:** 2026-08-13

**Authors:** Teppei Hashimoto, Kanji Tanaka, Yasuhiro Fujita, Takehiko Manabe, Hiroki Matsumiya, Masataka Mori, Masaru Takenaka, Koji Kuroda, Fumihiro Tanaka, Hidetaka Uramoto

**Affiliations:** Second Department of Surgery, Japan School of Medicine, University of Occupational and Environmental Health, 1-1 Iseigaoka, Yahatanishi- ku, Kitakyushu, Fukuoka 807-8556 Japan

**Keywords:** Non-small cell lung cancer, Neoadjuvant chemoimmunotherapy, Bulky N2 disease, Pathologic complete response, Aortic arch resection, Cardiopulmonary bypass, Case report

## Abstract

**Background:**

Neoadjuvant nivolumab plus chemotherapy increases pathologic complete response rates in resectable non-small cell lung cancer, but the operative safety of this approach in bulky, invasive N2 disease with suspected great-vessel involvement remains uncertain.

**Case presentation:**

A 64-year-old woman had left upper lobe adenocarcinoma with bulky single-station N2a disease and suspected aortic arch invasion. After three cycles of nivolumab plus carboplatin-paclitaxel, imaging showed only limited nodal shrinkage. At lobectomy, the subaortic lymph node was broadly adherent to the arch and surrounding structures, causing hemorrhage that required cardiopulmonary bypass and arch resection. Pathology showed pathologic complete response, but lymphocyte-predominant inflammation and fibrosis extended to the aortic adventitia.

**Conclusions:**

Radiographic response and pathologic complete response after chemoimmunotherapy do not necessarily indicate safe technical resectability in bulky, invasive N2 lung cancer. Multidisciplinary surgical planning should preserve caution when great-vessel invasion is suspected.

**Supplementary Information:**

The online version contains supplementary material available at https://doi.org/10.1186/s44215-026-00271-4.

## Background

Neoadjuvant immunotherapy combined with platinum-based chemotherapy has become an important option for resectable non-small cell lung cancer (NSCLC) after CheckMate 816 demonstrated higher pathologic complete response (pCR) rates than chemotherapy alone, with similar benefit reported in Japanese patients [1, 2]. However, these trials enrolled patients with already resectable disease and therefore do not address whether bulky or invasive N2 nodal disease can be safely resected, particularly when invasion of an adjacent great vessel is suspected.

Contemporary resectability statements for stage III NSCLC remain cautious: bulky N2 and invasive N2 disease are generally classified as unresectable or require exceptional multidisciplinary review [3–5]. Historically, resection involving the thoracic aorta has been limited to carefully selected patients and, when complete (R0) resection is achieved in T4N0-1M0 disease, has been reported as feasible with acceptable perioperative mortality and long-term survival [6–8]. We report a case in which pCR after neoadjuvant chemoimmunotherapy did not restore a safe dissection plane, because dense inflammatory adhesion persisted at the site of suspected aortic invasion despite the absence of viable tumor.

## Case presentation

**Fig. 1:**
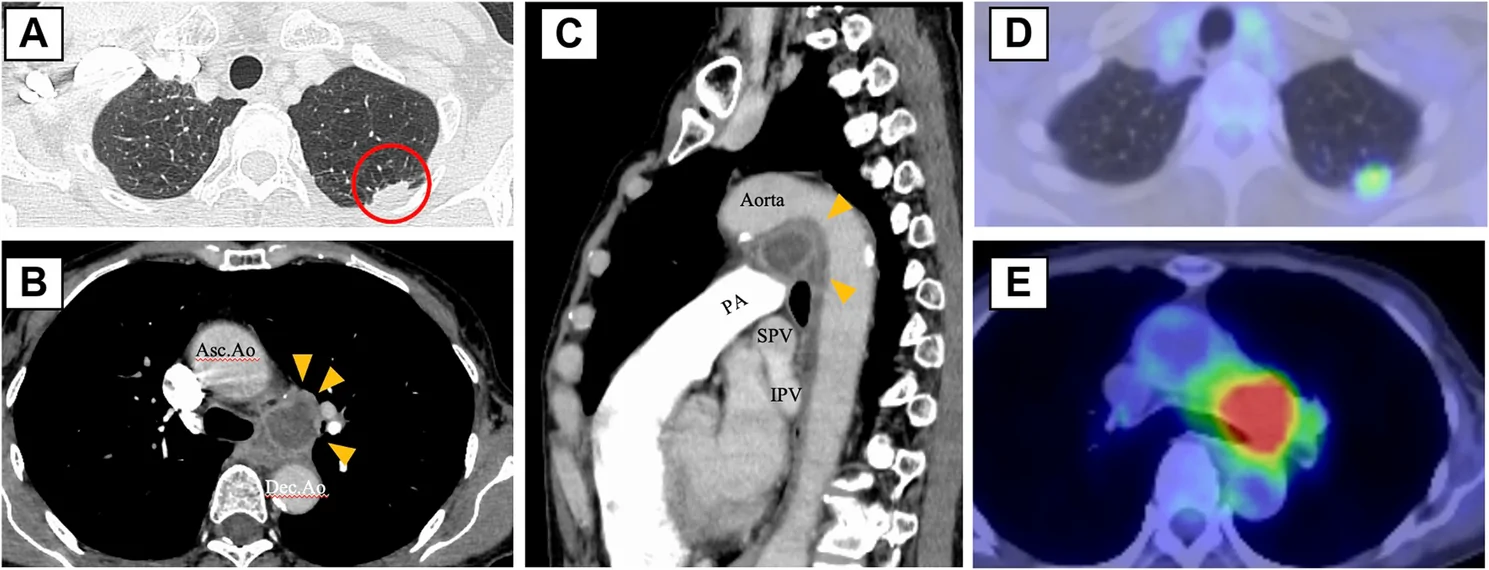
(**A**) Axial lung-window computed tomography (CT) demonstrates a 16-mm primary tumor in the left upper lobe (S1+2). (**B**) Axial and (**C**) sagittal mediastinal-window CT images reveal a bulky #5 lymph node abutting the aortic arch, with findings suspicious for direct invasion. (**D**, **E**) ¹⁸F-fluorodeoxyglucose (FDG) positron emission tomography (PET)/CT demonstrates intense FDG uptake in the primary tumor (**D**) and in the #5 lymph node (**E**), with no evidence of additional nodal involvement or distant metastasis

A 64-year-old woman was referred for hoarseness. Contrast-enhanced computed tomography (CT) showed a 16-mm solid tumor in the left upper lobe S1 + 2 and a bulky #5 lymph node with loss of the fat plane to the aortic arch, suggesting extranodal invasion. Fluorodeoxyglucose positron emission tomography-CT showed uptake in the primary tumor and #5 lymph node without other nodal or distant disease (Fig. 1). Attempted transbronchial needle aspiration of the #5 lymph node was unsuccessful because the inflammatory node was rigid. Thoracoscopic wedge biopsy of the left upper lobe lesion showed adenocarcinoma, negative epidermal growth factor receptor and anaplastic lymphoma kinase alterations, and programmed death ligand 1 expression of ≥ 75%. The clinical stage was cT1bN2aM0, stage IIIA, with single-station N2a disease, based on the 9th edition of the General Rule for Clinical and Pathological Record of Lung Cancer.

**Fig. 2:**
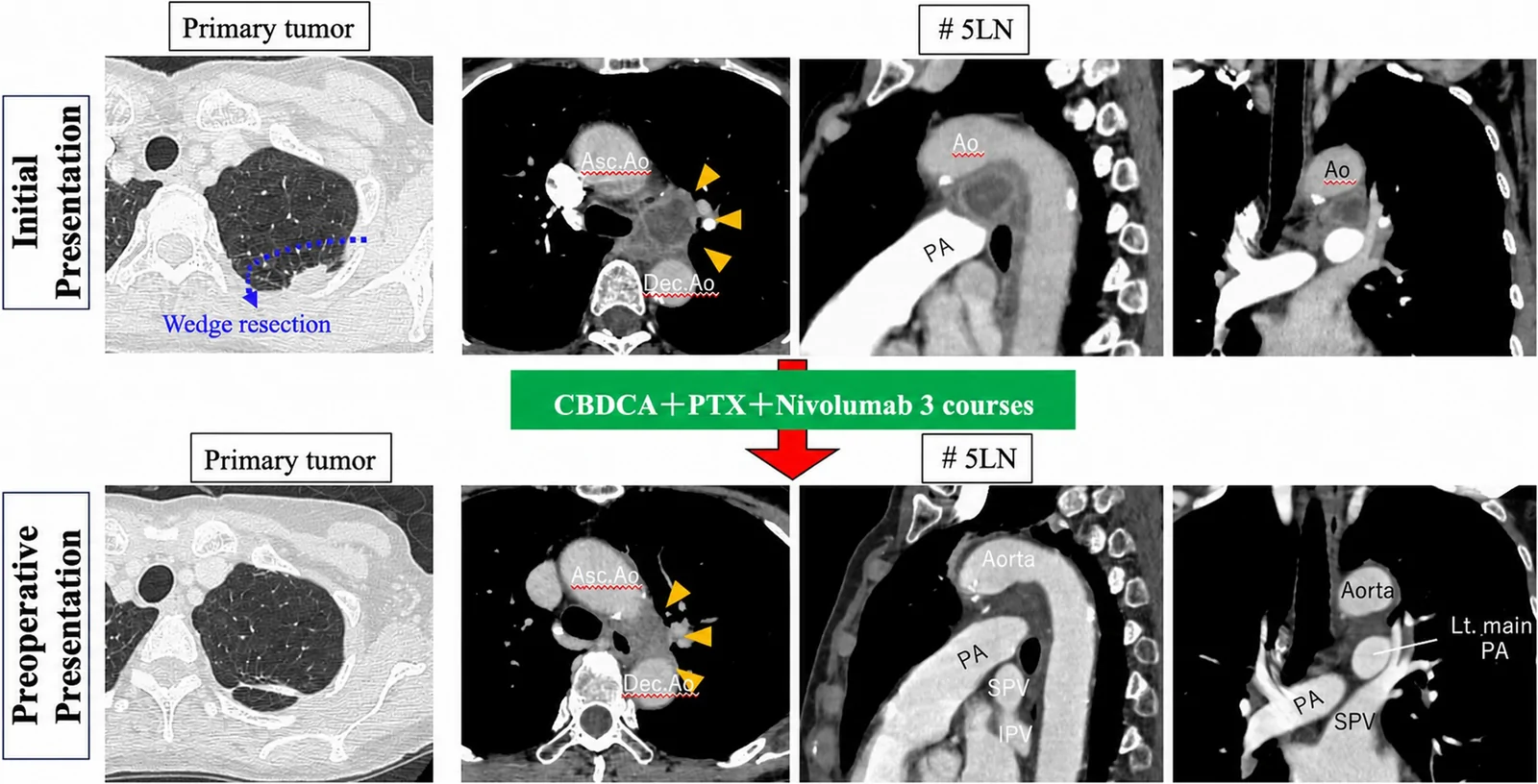
Treatment response and operative findings. After three cycles of nivolumab plus carboplatin-paclitaxel, no recurrence was seen at the wedge-resected primary site. The #5 lymph node showed slight shrinkage and reduced contrast enhancement, consistent with stable disease by RECIST

Neoadjuvant nivolumab plus chemotherapy was selected with curative resection as the intended strategy. She received carboplatin at area under the curve 5, paclitaxel 200 mg/m2, and nivolumab 200 mg/body for three cycles. No immune-related adverse event occurred. Restaging showed no recurrence at the biopsied primary site and slight shrinkage of the #5 lymph node, from 23 mm to 18 mm in short axis, representing a 14% reduction and stable disease by Response Evaluation Criteria in Solid Tumors (RECIST) (Fig. 2).

Although imaging demonstrated only stable disease and suspicion of aortic arch invasion persisted, our multidisciplinary tumor board recommended proceeding with curative-intent resection: there was no distant or nodal progression, and the disease remained single-station N2a and was judged potentially R0-resectable with cardiovascular backup. The patient was informed of the elevated operative risk, including possible great-vessel resection under cardiopulmonary bypass, and provided consent. The patient was placed in a modified right lateral decubitus (“corkscrew”) position, allowing simultaneous access to the left thorax and the femoral vessels for cardiopulmonary bypass cannulation if required. Surgery was performed through left posterolateral thoracotomy with cardiovascular backup arranged before incision; aortic arch replacement under cardiopulmonary bypass (CPB) was planned if arch invasion by the #5 lymph node was confirmed. The main pulmonary artery and superior pulmonary vein were controlled intrapericardially. In contrast, the #5 lymph node was broadly and densely adherent to the aortic arch, left recurrent laryngeal nerve, and surrounding mediastinal tissues. During attempted dissection over the aorta, catastrophic bleeding occurred from the aortic arch at the suspected invasion site before planned vascular control (Additional file 1). Immediate manual compression was applied but failed to control the hemorrhage; we therefore proceeded to identify the bleeding site under CPB support. An arterial return cannula was placed in the right femoral artery and a venous drainage cannula in the inferior vena cava, and CPB was established. To identify the bleeding point, the core temperature was lowered to 21 °C and total circulatory arrest was induced. After circulatory arrest, the injured aortic arch was found to be dehisced over approximately half of its circumference, and selective cerebral perfusion was promptly instituted (Additional file 2). Left upper lobectomy with ND2a-1 dissection, en bloc resection of the #5 lymph node and aortic arch, and graft reconstruction were completed (Additional file 3). Operative time was 725 min, and blood loss was 8389 mL.

**Fig. 3:**
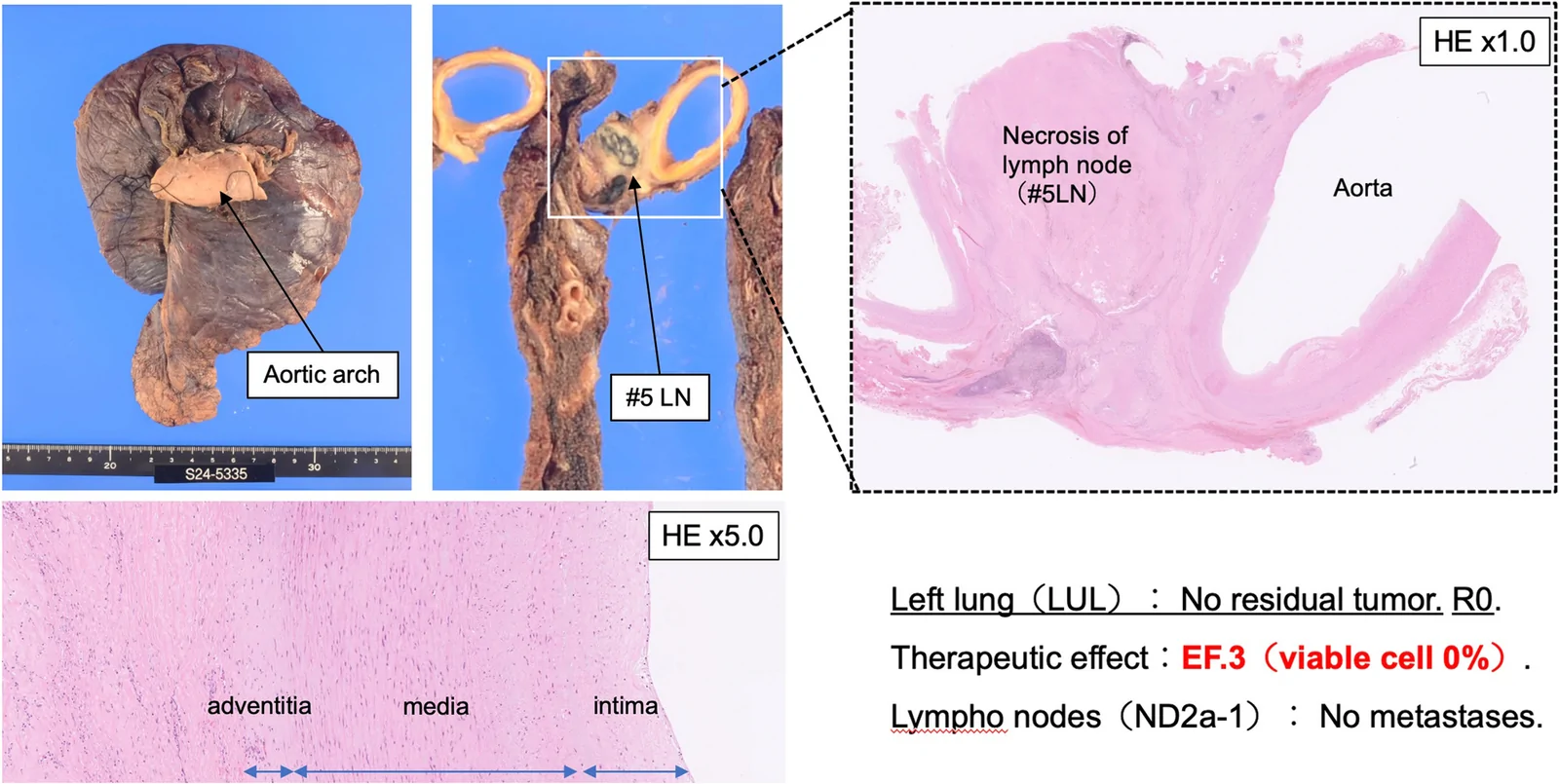
Histopathology after chemoimmunotherapy. No viable tumor remained in the lung or lymph nodes, consistent with pathologic complete response. Hematoxylin and eosin staining showed lymphocyte-predominant inflammatory-cell infiltration extending to the aortic adventitia and continuous with surrounding stroma. Original magnifications: low-power view and x5

Pleuritis on postoperative day 9, classified as Clavien-Dindo grade IIIa, improved after thoracentesis, and the patient was discharged on day 20 without neurologic sequelae. Histopathology showed no viable residual tumor in the lung or lymph nodes, consistent with pCR, 0% viable tumor, Ef.3, and R0 resection. The aortic-wall specimen showed lymphocyte-predominant inflammatory-cell infiltration extending to the adventitia and becoming continuous with surrounding stroma (Fig. 3). The patient remains alive without recurrence 22 months after surgery.

## Discussion

This case highlights a practical gap between oncologic response and technical resectability in bulky, invasive N2 disease after chemoimmunotherapy. In retrospect, the limited nodal shrinkage—a 14% reduction corresponding to stable disease by RECIST—together with the persistent preoperative suspicion of aortic involvement were themselves indicators of a technically demanding operation. The key limitation was therefore not that imaging failed to signal operative risk, but that current imaging modalities cannot reliably distinguish viable tumor from treatment-related fibrosis, nor accurately define the surgical dissection plane. The pathologic findings illustrate this: even after pCR, lymphocytic inflammation and fibrosis extended to the aortic adventitia, leaving a hazardous mediastinal interface rather than a restored dissection plane.

The case therefore supports existing caution in resectability assessment. Consistent with the recent multisocietal consensus on the technical resectability of stage III NSCLC, bulky and invasive N2 disease carries a high operative risk and should generally be regarded as unresectable [5]. When bulky and invasive N2 disease is accompanied by suspected extranodal extension to the aorta, the risk is not only whether viable tumor remains but also whether treatment-related or tumor-related fibrosis has fixed the node to critical structures. Radiographic response, stable disease, or eventual pCR should not be interpreted as evidence that dissection will be technically safe, because current imaging cannot resolve whether persistent soft tissue at the great-vessel interface represents viable tumor or treatment-related fibrosis, nor delineate the plane available for safe dissection.

For similar cases, persistent loss of the fat plane or aortic contour deformation on restaging should be treated as warning signs of a potentially inseparable interface irrespective of RECIST response [9]. Dedicated arterial-phase CT angiography can map arch-branch anatomy, vascular access, and potential clamping or endograft landing zones; cine MRI may add information when CT is equivocal [10, 11]. Neither modality has been validated to distinguish viable tumor from post-chemoimmunotherapy fibrosis, particularly fibrosis from a #5 node, or to demonstrate a safe dissection plane. Multidisciplinary planning should therefore convert cardiovascular “backup” into a predefined control strategy specifying exposure, proximal and distal control, cannulation readiness, and criteria for abandoning subadventitial peeling. Limited involvement in a favorable location may permit tangential or direct clamping and patch repair, whereas extensive arch involvement may require planned CPB with hypothermia and cerebral protection [6, 12].

Preoperative or same-session thoracic endovascular aortic repair (TEVAR) may provide endoluminal protection and facilitate en bloc aortic-wall resection without CPB [11, 13]. Selection requires suitable access, adequate disease-free landing zones, and planned preservation or revascularization of supra-aortic branches, making TEVAR less straightforward at the arch than at the descending aorta. This off-label oncologic use is supported only by small retrospective series; risks include endoleak, branch-vessel injury, stroke, spinal cord ischemia, graft migration or herniation, and infection [13, 14]. TEVAR should therefore be considered case by case by an experienced aortic-thoracic team, not routinely. If safe vascular control and R0 resection cannot both be anticipated, definitive nonsurgical therapy remains appropriate.

Preoperative planning should assume that vascular injury may occur before planned vascular control can be achieved, even when cardiovascular backup has been arranged. The main lesson is that pCR after neoadjuvant chemoimmunotherapy does not ensure safe technical resectability in bulky, invasive N2 lung cancer with suspected great-vessel involvement.

## Supplementary Information

Additional file 1. Operative video 1. Operative video showing the surgical positioning (modified right lateral decubitus "corkscrew" position) and the operative approach through to hemorrhage from the aortic arch at the suspected invasion site, with broad, dense #5 nodal adhesions to the aortic arch and surrounding structures.

Additional file 2. Operative video 2. Operative video showing establishment of cardiopulmonary bypass through dissection around the #5 lymph node.

Additional file 3. Operative video 3. Operative video showing resumption of spontaneous cardiac activity, pulmonary artery angioplasty, and combined left upper lobectomy with aortic arch resection.

## Data Availability

All data supporting the findings of this case report are included within the article and its supplementary information files. Additional clinical details are available from the corresponding author on reasonable request, subject to patient privacy protections.

## Abbreviations

CPB: Cardiopulmonary bypass
CT: Computed tomography
NSCLC: Non-small cell lung cancer
pCR: Pathologic complete response
RECIST: Response Evaluation Criteria in Solid Tumors
TEVAR: Thoracic endovascular aortic repair
